# Advances in symptomatic therapy for left ventricular non-compaction in children

**DOI:** 10.3389/fped.2023.1147362

**Published:** 2023-05-04

**Authors:** Dong Li, Ce Wang

**Affiliations:** Department of Pediatrics, Shengjing Hospital of China Medical University, Shenyang, China

**Keywords:** left ventricular non-compaction (LVNC), therapy, children, myocardiopathy, heart failiure

## Abstract

Left ventricular non-compaction is a complex cardiomyopathy and the third largest childhood cardiomyopathy, for which limited knowledge is available. Both pathogenesis and prognosis are still under investigation. Currently, no effective treatment strategy exists to reduce its incidence or severity, and symptomatic treatment is the only clinical treatment strategy. Treatment strategies are constantly explored in clinical practice, and some progress has been made in coping with the corresponding symptoms because the prognosis of children with left ventricular non-compaction is usually poor if there are complications. In this review, we summarized and discussed the coping methods for different left ventricular non-compaction symptoms.

## Introduction

With the advancements in diagnosis and treatment techniques, the detection rate of myocardial insufficiency in children has increased. Left ventricular non-compaction (LVNC) is a rare and complex condition in children with cardiomyopathy. It is characterized by the presence of thick and reticulated myocardial trabeculae in the ventricular cavity with deep, sunken fossae connecting to the left ventricular cavity. In recent Australian retrospective studies, the incidence of LVNC in children was reported to be approximately 0.11/100,000 (1), and several investigations demonstrated that the majority of pediatric patients with LVNC have a poor prognosis, especially those with comorbidities other than cardiomyopathies (1–4).

At present, LVNC is the most commonly used diagnostic method to detect the morphological characteristics of left ventricle by echocardiography. In the past cohort studies, specific echocardiographic criteria were established, including Jenni, Chin and Stollberger (5–7). And the widely used LVNC diagnostic criteria are mainly Jenni diagnostic criteria. Mainly in these aspects: (a) it includes dense layer and non-dense layer, and the ratio of the thickness of non-dense layer myocardium to the thickness of dense layer myocardium is more than 2 (children are more than 1.4), so it is necessary to pay attention to the measurement time in systole. (b) The lesion area is generally located at the apex of the heart (>80%), and some patients’ lateral walls and inferior walls will be involved. (c) Color Doppler can see that there is blood flow communication, but it should be noted that blood flow is not connected with coronary circulation.

It is worth mentioning that, in recent studies, LVNC is not usually recognized and LV hypertrabeculation is perhaps more accurate (8). This is because in studies of some adult cases, it has been found that the degree of myocardial trabecular densification varies from one person to another and from one site to another in the same individual. For some adult myocardial tissue, the degree of myocardial non-dense on imaging may meet the diagnostic criteria, but the patient does not show any symptoms. In addition, myocardial nondensification is usually accompanied by other cardiac diseases such as dilated cardiomyopathy or hypertrophic cardiomyopathy, but there is no clinical evidence that their outcome and prognosis are affected by the degree of myocardial nondensification. When these patients are diagnosed with LVNC, perhaps overdiagnosis is involved, and this may be normal in some patients, such as pregnant women or athletes. Therefore, the use of LV hypertrabeculation, more inclined to describe the imaging phenotype, is more recommended in studies than easily as a clinical diagnosis.

However, in pediatric cases, the use of LV hypertrabeculation in children still requires caution due to the paucity of relevant studies and the fact that, as a congenital heart disease, it is not easy to determine differences in the degree of myocardial trabecular densification in children given the relatively short time of cardiac development in children compared to adults. In this article, we tentatively follow the diagnosis of LVNC because of the inability to obtain strong support.

The clinical symptoms of LVNC in children are complex. LVNC may be clinically asymptomatic or present with a variety of symptoms such as chest pain, dyspnea, and palpitations; however, three main clinical symptoms require urgent attention (9–12). The most common and the most important is heart failure, which is associated with most of the other clinical symptoms (13–16). Thromboembolism and arrhythmias are also common complications of high clinical concern in patients with LVNC. In addition, these patients often have a neuromuscular disease and may experience fatigue (17, 18), muscle aches and pains, and elevated creatine kinase levels (19). The relevant data are compiled in Table 1. Moreover, even though several children with LVNC have adverse outcomes, to date, no clinically targeted treatment exists, and only symptomatic or prophylactic treatment is available (20–23).

**Table 1:** Clinical findings in pediatric patients with isolated non-compaction cardiomyopathy.

Pediatric patients	Ichida F et al. (7)	Wald R et al. (24)	Koh C et al. (8)	Brescia ST et al. (25)	Wang C et al. (21)		van Waning JI et al. (12)	Shi WY et al. (1)	Sabatino J et al. (9)
					Infanitile type	Juvenile type			
Number of patients	27	22	10	242	108	97	52	29	23
Male patients, *n* (%)	15 (56%)	40%	7 (70%)	145 (60%)	59 (55%)	51 (53%)	—	20 (69%)	12 (52%)
Age range, years	1–15	—	0.019–12	0.3–13.9	0–1	1–15	0–15	0.08–1.3	0–18
Length of follow-up (median)	17 years	16 years	8 years	4 years	3.5 years	5.9 years	4.2 years	6.8 years	5.4 years
Age at diagnosis (median)	—	3.9 years	—	7.2 ± 6.9 years	2.7 months	7.3 years	—	0–1 years	—
Family history of cardiomyopathy	12 (44%)	18%	—	33 (14%)	37 (34%)	36 (37%)	8 (15%)	9 (31%)	3 (13%)
Death in follow-up	2 (7%)	14%	3 (30%)	31 (13%)	14 (13%)	9 (10%)	8 (15%)	14 (48%)	—
Clinical characteristic (at the time of diagnosis)									
Asymptomatic	—	—	1 (10%)	89 (37%)	21 (19%)	52 (54%)	9 (17%)	—	—
Heart failure	6 (22%)	54%	8 (80%)	60 (25%)	65 (60%)	22 (23%)	13 (27%)	24 (83%)	—
Arrhythmia	6 (22%)	—	3 (30%)	80 (33%)	9 (8%)	11 (11%)	9 (17%)	—	2 (9%)
Thromboembolism	2 (7%)	—	0(0%)	—	5(5%)	5(5%)	2(4%)	—	0(0%)

Generally speaking, left ventricular noncompaction is a congenital disease with unknown etiology (26). At present, there is no literature to prove that adult myocardial noncompaction has an acquired trend or mechanism. Some patients with left ventricular noncompaction are asymptomatic from birth to onset, and it is not discovered until they have heart-related symptoms or physical examination. This is called myocardial noncompaction in adults. Therefore, both adults and children with myocardial noncompaction are congenital diseases, but the time of discovery or symptoms is different (27).

**Figure 1:**
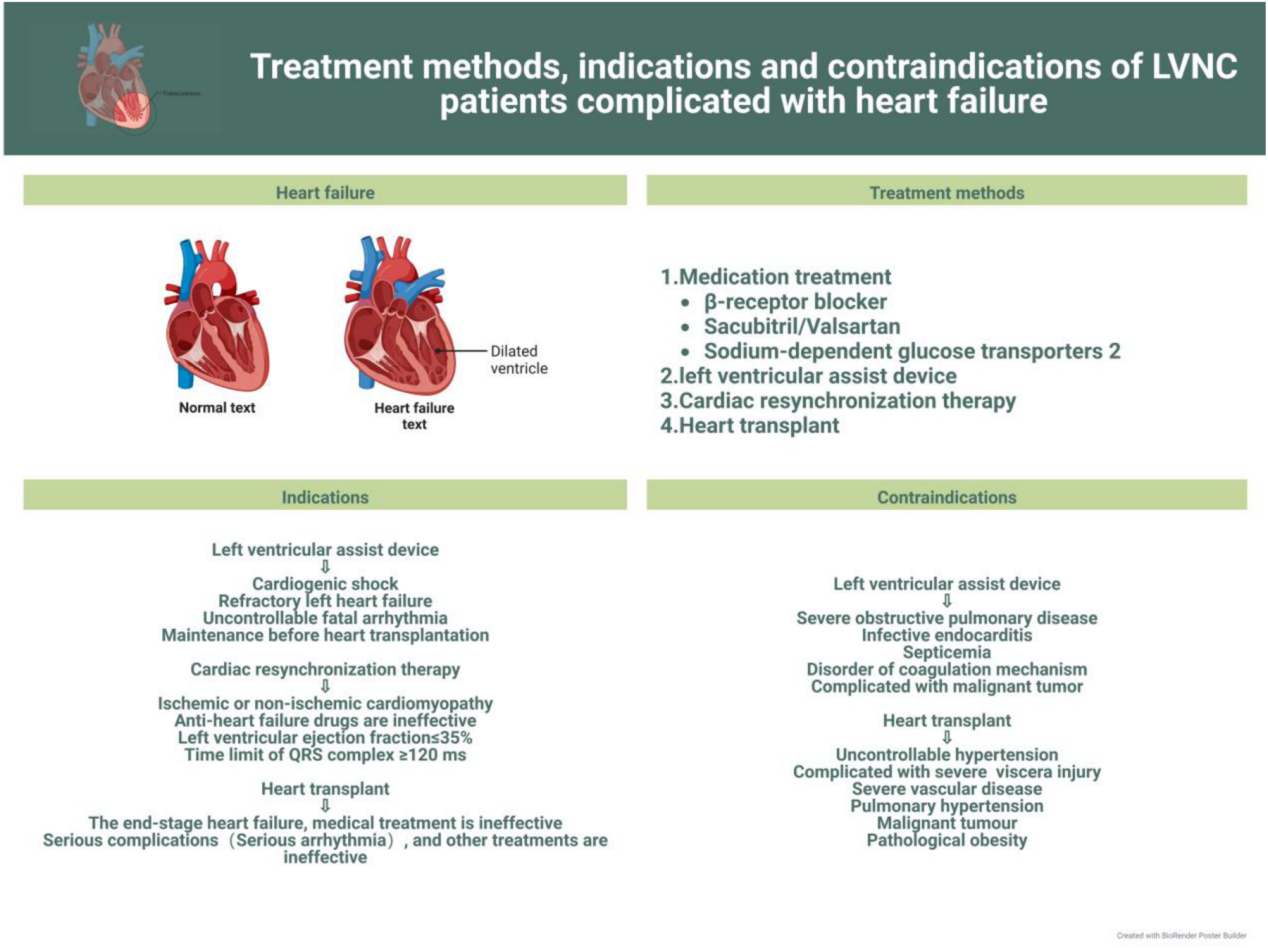
Treatment of LVNC complicated with heart failure.

**Figure 2:**
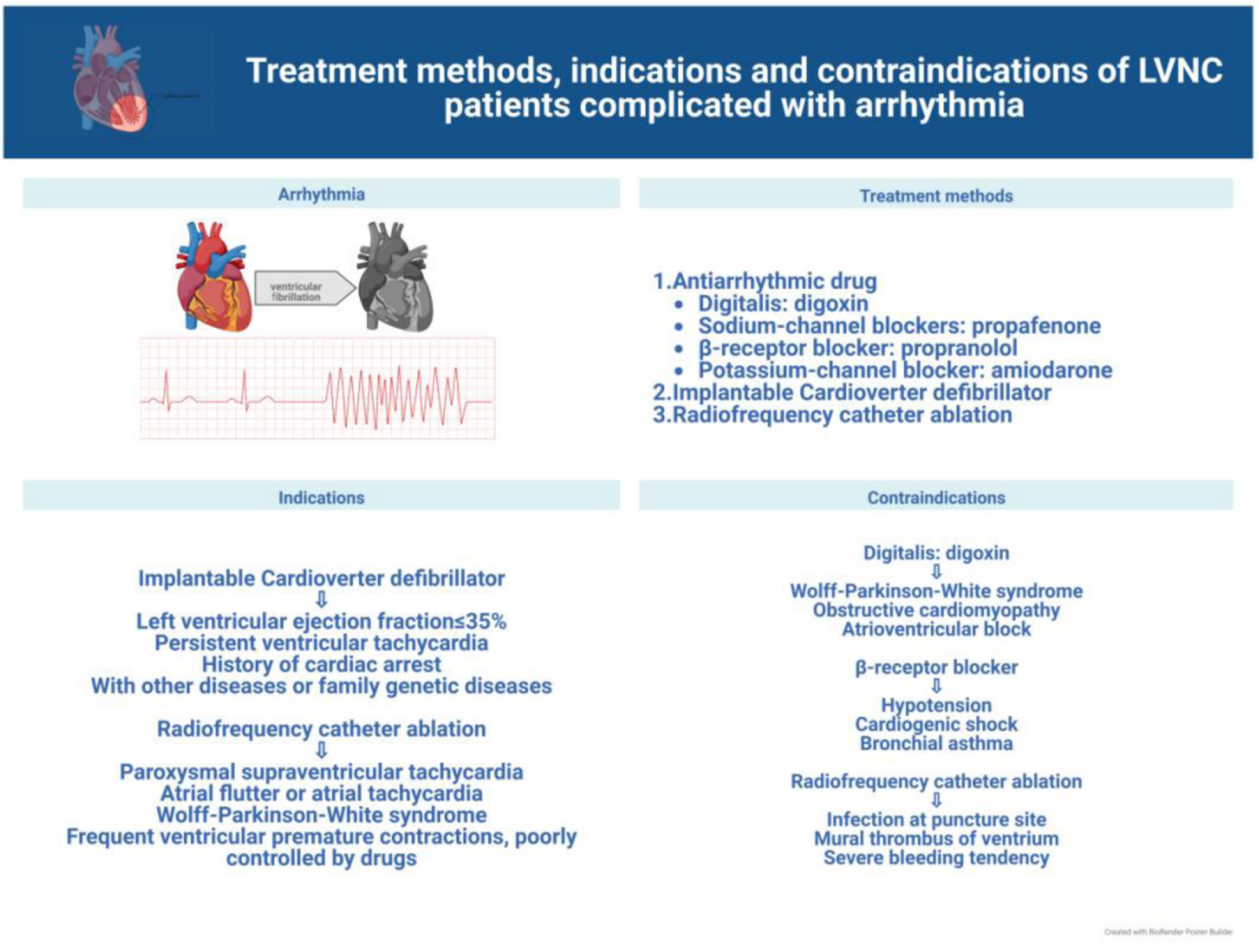
Treatment of LVNC complicated with arrhythmia.

**Figure 3:**
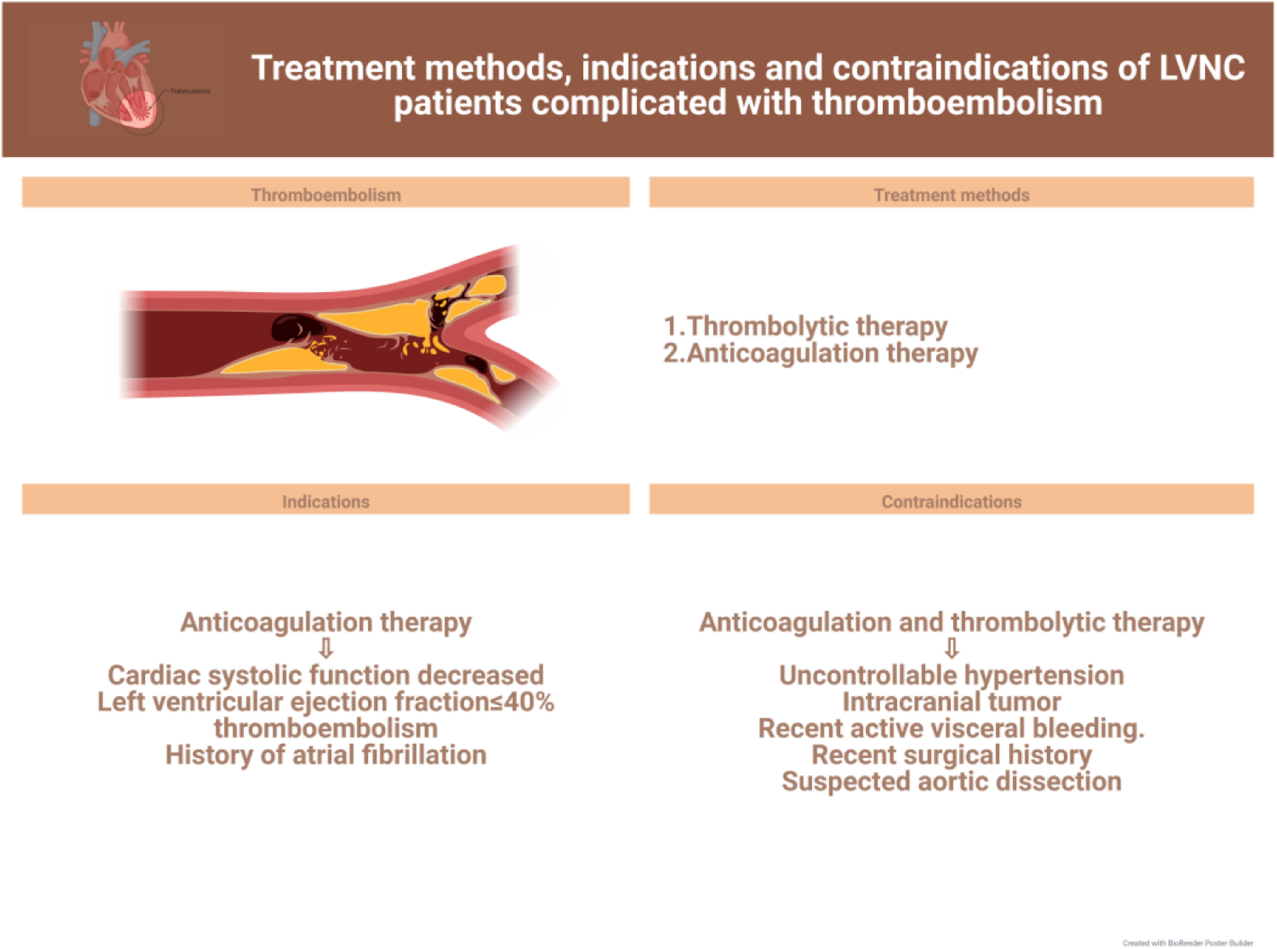
Treatment of LVNC complicated with thromboembolism.

In this review, we discuss current advances in the clinical management of the different symptoms of LVNC to further the search for more effective treatments for the various related complications and facilitate the progress of clinical research. The latest treatment strategy, indications and contraindications are compiled in Figures 1–3. Simultaneously, our review provides potential insight for clinical discoveries in the treatment of LVNC.

## Heart failure

Patients with LVNC can show many symptoms or no symptoms. To be sure, LVNC can lead to heart failure (9, 15). However, according to the researchers’ data, among LVNC patients, patients with heart failure symptoms have worse prognosis and higher mortality than those with isolated LVNC (28, 29). However, some researchers think that heart failure is not a high-risk factor for LVNC (1, 30, 31). Under any circumstances, prevention and treatment of heart failure should be prioritized (32) (Figure 1).

However, prevention and treatment of heart failure in children still require improvements. The etiology of heart failure in children, usually caused by congenital heart disease, is different from that in adults, which is usually ischemic. Therefore, the therapeutic management of heart failure in children also differs from that in adults in some respects. The onset of heart failure in children is usually associated with symptoms such as fatigue, shortness of breath, exercise intolerance, and sometimes more serious symptoms, therefore necessitating prompt and effective treatment. There are two widely accepted clinical principles of treatment: elimination of the cause and control of symptoms and disease progression (33, 34). The primary goal of managing children with heart failure is to closely monitor their general condition and arrange nutritional support rationally. For children with LVNC, close attention should be paid to changes in oxygen partial pressure and provide ventilation support if necessary. Additionally, digoxin should be administered to enhance myocardial contractility in children with LVNC and left heart systolic dysfunction. Other clinically recognized and recommended drugs include β-blockers and those of the ACEI class (33, 35, 36).

Ivabradine is a selective inhibitor of sinus node If current, which can specifically reduce the heart rate, but has no obvious effect on cardiac conduction time, myocardial contractility and ventricular repolarization (37). Clinical studies show that heart rate is significantly related to the prognosis of heart failure, and ivabradine can reduce the hospitalization rate and mortality rate of HF (25, 38, 39). Many authoritative heart failure guidelines strongly recommend HF (heart failure with reduced ejection fraction) for adults as Class IIa (38, 40–42), and pediatric heart failure guidelines are recommended as promising drugs for HF(chronic heart failure (CHF) in children (43). At present, only tablets are approved for adult CHF in China, and the Food and Drug Administration (FDA) has supplemented and approved oral liquid and tablets for children with stable HF caused by DCM for 6 months and above, which provides evidence-based evidence for clinical use in pediatrics. Specific usage and dosage are as follows: (1) for children over 6 months old and weighing less than <40 kg, the initial dosage is 0.05 mg/kg, twice a day, taken with meals, and the dosage is adjusted every two weeks according to the tolerance to reduce the heart rate by at least 20%; the maximum dose is 0.2 mg/(kg/times) (children aged 6 months to <1 year) or 0.3 mg/(kg/times) (children aged ≥1 year), and the total dose does not exceed 7.5 mg/time. (2) Children with body weight ≥40 kg: the initial dose is 2.5 mg, twice a day, and the dose is adjusted every two weeks according to the tolerance to reduce the heart rate by at least 20%, and the maximum dose is 7.5 mg/time (44).

Phase II/III clinical studies show that ivabradine can safely and effectively reduce the resting heart rate of children, and the left ventricular ejection fraction, clinical cardiac function classification and quality of life have a good improvement trend (24). At the same time, because it is dose-dependent, its activity depends on the opening and closing of If current channel, which can reach saturation state and prevent the adverse events of infinite decrease of heart rate (45, 46). It provides a new drug treatment idea for children with heart failure who still have symptoms, reduced left ventricular ejection fraction (LVEF), sinus rhythm and resting heart rate of not less than 70 beats/min after using traditional anti-heart failure drugs simultaneously, and can be used as an alternative. At the same time, considering its characteristics of direct action on sinus node and few adverse reactions, it has a good application prospect for the treatment of heart failure in children with sinus tachycardia.

Long-term use of β-blockers can improve the symptoms and quality of life of adults with heart failure, and reduce the risk of death and hospitalization. However, children's studies show that carvedilol can improve the echocardiographic parameters and serum brain natriuretic peptide (BNP) level to some extent, but it has only a tendency to improve the prognosis of clinical heart failure (47–49). Therefore, there is not enough data to recommend or prevent it for children with congestive HF.

Some studies demonstrate the effectiveness of using drugs such as sacubitril/valsartan and SGLT2 inhibitors for the treatment of heart failure (34); however, this drug has only been proved to be applicable to the treatment of heart failure in adults, and no study has proved that this drug can be applied to children. Therefore, the safety and effectiveness of this drug in children patients are unknown. Besides, although the research on the efficacy of diuretics in pediatric HF population is limited at present, it can be confirmed that diuretics play an important role in the acute management of symptomatic HF patients. Through the review of adult diuretic treatment, it is confirmed that the use of diuretics can effectively alleviate symptoms, reduce the onset of HF deterioration and improve living conditions (50). Through these data and empirical evidence, it is enough to prove that their routine use in the emergency of HF children is reasonable. Diuretics can reduce the fluid accumulation in children and reduce the burden on the heart (51).

However, although these studies suggest that their use in patients with heart failure is warranted, it is unknown whether they can improve the condition of patients with LVNC. Therefore, after clinical trials and evaluation, these drugs may be promising (52, 53).

Although pharmacological therapy can significantly improve the quality of life in children with heart failure, there is still a large proportion of children with poor prognosis owing to disease progression or other factors; therefore, these children should be considered for cardiac assist device implantation or even heart transplantation (33, 54). The implantation of a left ventricular assist device (LVAD) has been shown to be effective when medications fail to improve LV systolic and diastolic function in patients with LVNC (55). In an Italian study (56), researchers concluded that cardiac resynchronization therapy(CRT) was effective in improving LV function in 52 heart failure volunteers (20 with DCM plus LVNC and 32 with DCM alone) using cardiac resynchronization therapy and was more effective in patients with LVNC than in those with DCM alone. The efficacy becomes more evident with a larger area of myocardial densification. Heart transplantation as the ultimate treatment must be beneficial for patients with LVNC or even other cardiomyopathies (57, 58), but few cases of heart transplantation have been performed. Mexico reported the first case of a 20-year-old LVNC patient who underwent heart transplantation (59), and the outcome was successful within 15 months with no acute rejection on intramyocardial biopsy. Its long-term prognosis needs further follow-up investigation (60–63).

## Arrhythmia

Arrhythmias in patients with LVNC usually symptomatically manifest as weakness and palpitations (13, 14) and require attention and effective treatment (20, 64). All patients with LVNC who develop arrhythmias should be routinely treated with antiarrhythmic drugs. In a limited pediatric cohort study, the use of β-blockers et al. antiarrhythmic drugs was found to reduce left ventricular ejection fraction and volumes significantly in patients with LVNC (35, 65), with carvedilol having effectively improved left ventricular function; however, the long-term efficacy is unclear (66). Sotalol has been proved to be effective in the treatment of ventricular arrhythmia and atrial fibrillation in both adults and pediatrics (67, 68). It is worth mentioning that antiarrhythmic drugs should be used with caution because of their side effects and unknown risks (35, 69, 70) (Figure 2).

In addition, the use of implantable cardiac defibrillators can effectively prevent ventricular tachycardia and sudden death (71–73). Implantation is especially indicated when at least one of the following conditions is met: left ventricular ejection fraction ≤35% (74), sustained ventricular tachycardia or previous cardiac arrest (75), presence of comorbidities or family history of genetic disorders (76). Risk assessment by analyzing the ECG characteristics of patients with cardiomyopathy has also been performed to determine the risk of ventricular tachycardia (77) for early detection and prevention, and to provide some guidance for the use of implantable cardiac defibrillators.

A direct and effective approach for the treatment of arrhythmias is catheter radiofrequency ablation (60, 78), which has great treatment advantages as the procedure is easy to perform, does not cause damage to the heart, and is minimally invasive, while the patient bears minimal pain and recovers quickly. However, some recent investigations have found arrhythmogenic lesions in the epicardial tissue, requiring the endocardium and epicardium to be operated (19, 36, 79). In the study by Sohns et al., 10 of 18 patients with LVNC underwent catheter radiofrequency ablation (two of them underwent endocardial and epicardial ablation) with a 90%success rate. After follow-up, the mortality rate of patients who did not undergo radiofrequency catheter ablation was approximately three times higher than that of patients who underwent radiofrequency catheter ablation; thus, it was concluded that radiofrequency catheter ablation is safe and effective for patients with LVNC (71). In addition, catheter radiofrequency ablation is contraindicated in patients with wall thrombus in the ventricular cavity, which may lead to dangerous thrombus dislodgement.

## Thromboembolism

Owing to the presence of myocardial trabecular gaps in LVNC patients, there is a high risk of thrombus formation during blood flushing (36, 80). Statistically, the risk of thrombosis in patients with LVNC is about 21%–38% (52, 81). Thrombosis can be a fatal threat as it may cause complications such as stroke, pulmonary embolism, and mesenteric ischemia (Figure 3).

It is well known that anticoagulation and thrombolytic therapy are clinically applied for patients with LVNC who have thrombosis. However, the need for prophylactic anticoagulation in these patients is still controversial. Although there is a lack of prospective studies to make predictions, according to some recent studies and current medication guidelines (9, 82), prevention of thromboembolic complications is a clinical priority and all patients with LVNC require routine prophylaxis against thromboembolism (83, 84). In a 30-month investigation on a cohort including 17 patients with LVNC, Ritter et al. found that the incidence of thromboembolism was approximately 24%; therefore, the researchers concluded that thromboembolism occurs independent of left ventricular function and size, and that LVNC itself is a high-risk factor for it. Ultimately, the researchers supported anticoagulation for all patients with LVNC (84).

Anticoagulation is not necessary in asymptomatic patients or those with normal cardiac function and it even increases patient burden. In a mean follow-up of 229 patients with LVNC without AF by Fazio et al., the incidence of thromboembolic events was only 2.1% (85). Instead, in more severe cases, other studies found prophylactic anticoagulation in patients with heart failure to increase the risk of bleeding (86, 87).

Nonetheless, anticoagulation is clinically mandatory when LVNC patients have reduced cardiac systolic function, an ejection fraction below 40%, or thromboembolism or previous atrial fibrillation (88–90). Since the risk of thrombosis is substantially increased in patients with LVNC due to the deep grooves between myocardial trabeculae, prophylactic anticoagulation is not a problem in asymptomatic patients, although the risk of bleeding due to anticoagulation therapy cannot be ignored. In clinical practice, patients are treated according to their needs and the corresponding guideline criteria. In general, the CHADS2/CHADS2-Vasc score is commonly used as a medication guideline to analyze the risk of thromboembolism in children with LVNC (26, 91).

Unfortunately, in all anticoagulant therapy strategies, even within the normal treatment range, bleeding is inevitable, which is also a major complication of anticoagulant therapy (92, 93). The risk level of bleeding can refer to Spyropoulos’ research (94). When bleeding occurs during anticoagulation, there is no way to prevent it. When bleeding occurs during anticoagulation, the location, cause and severity of bleeding should be evaluated as quickly and accurately as possible, and specific treatment should be given, including mechanical pressing and lowering the dose of anticoagulant (95). When massive bleeding occurs (the standard is that the bleeding is serious enough to require major medical intervention, such as blood transfusion or surgery, and the prognosis is extremely poor) (92, 96), generally speaking, it is necessary to stop anticoagulation treatment immediately, quickly evaluate the degree of bleeding and life state of the patient, use specific reversal agents to reverse the anticoagulation effect, and use mechanical ventilation and blood transfusion to maintain life if necessary (97–100). As long as the patient has bleeding or bleeding risk during anticoagulation, it is necessary to closely detect the patient's life state, maintain the patient's body temperature and closely detect the patient's blood gas ion stability. However, if careful anticoagulation is carried out according to the known bleeding risk factors, the risk of bleeding will be greatly reduced (101).

In addition, recent clinical case reports have shown (27) that a 65-year-old patient with LVNC in Japan suffered from worsening symptoms such as dyspnea and heart failure. After being hospitalized in an emergency department, the patient was found to have cerebral infarction. After establishing a perfect cardiopulmonary bypass, a left ventricular incision was made to remove the thrombus in the ventricle. At the same time, the protruding muscle trabecula in the patient's left ventricle was removed as much as possible. After the operation, the patient's left ventricular diastolic function and left ventricular ejection fraction were gradually improved within one year, and serious symptoms such as heart failure could be effectively solved by eliminating the cause. Therefore, excision of prominent trabeculae may be effective in improving symptoms of LVNC patients (102–104), which also provides a novel direction for the treatment of LVNC; however, its long-term prognosis still needs further follow-up.

## Prospective treatment

Gene mutations, such as those involving sarcomere and ion channel genes/proteins, can lead to LVNC (91). It has been demonstrated (105) that LVNC can be induced in the mouse heart using excess All-Trans Retinoic Acid. The successful establishment of this animal model provides a completely new platform for exploring potential LVNC therapeutic approaches in the future. In 2007 Takahashi et al. (106) successfully generated iPSCs cells from adult human dermal fibroblasts. It provides a basis for cultivating LVNC animal models (107). Moreover, recent studies are gradually devoted to constructing iPSCs model to further explore the pathways that may be related to the pathogenesis and treatment direction of LVNC. For example, some researchers have successfully established an LVNC-derived IPS cell model, and detected that the expression level of RhoA protein, a key protein of Rho pathway, is up-regulated and the degree of phosphorylation is significantly increased in the disease group (abnormal cytoskeleton changes) by Western blot, suggesting that this change may be related to Rho/ROCK pathway. Researchers believe that the imbalance of cytoskeleton and polarity may be involved in the occurrence of myocardial noncompaction, and the change of Rho/ROCK pathway activity may be its potential pathogenesis. Although there is not a lot of data to support it, it still provides a new idea for the treatment of LVNC (108).

The implementation of gene editing technologies, including zinc finger nucleases (ZFNs), transcription activator-like effector nucleases (TALENs), and clustered regularly interspaced short palindromic repeats (CRISPR) systems (109), has made the generation of LVNC cardiomyocytes a reality. Gene editing techniques have been widely utilized in cardiomyopathy research and there have been attempts to explore the role of various genes in the pathogenesis of LVNC (110, 111). There have been successful experiments using TALENs technology to introduce MYH7 mutated genes into a pig model to obtain a HCM model (112). Further, the ability to obtain LVNC animal models provides greater opportunities for treatment experimentations, including the possibility of preventing or eliminating LVNC by means of gene knockout or mutant gene repair, which should be gradually considered (113). However, the effectiveness of genetic strategies to treat LVNC and ethical considerations warrant further discussion and reviewing.

## Prevention and restrictions on daily activities

In addition to the distinct symptomatic treatment described above, another potential strategy is worth exploring for patients with LVNC. It is well known that patients with symptomatic LVNC have much higher mortality and worse prognosis compared to those with an asymptomatic presentation (74, 114), especially in patients with concomitant heart failure. Although controlling LVNC symptoms’ manifestation is impossible, its diagnosis before symptom development can effectively improve quality of life. In a retrospective cohort study in Japan (31) from 2000 to 2017, 44 of 105 pediatric LVNC patients (41.9%) were identified during school screening, and most of these students exhibited abnormal QRS wave segments on the electrocardiogram (ECG). With the detection of ECG abnormalities, school screening may be an important factor in the detection of patients with LVNC in the future (Table 1).

In addition, genetically screening families of patients with LVNC, may also elucidate whether LVNC is hereditary and determine what changes in their family's related gene is responsible and what effects this change might bring to their relatives, which could be important for future LVNC eradication and prevention (20, 115). According to statistics, the proportion of LVNC patients with family history is tremendous (Table 1).

Patients with LVNC can potentially engage in moderate physical activity (60, 116); this in turn can help prevent cardiovascular disease, as stated in the 2020 ESC Guidelines on sports cardiology and exercise in patients with cardiovascular disease (117); the section for patients with LVNC states that only individuals with LVEF >50% and without arrhythmias should engage in high-intensity physical activity as well as competitive sports, and only individuals with LVEF >40% should engage in appropriate physical activity. However, the expected outcome requires further clinical investigation.

## Perspectives and conclusion

Although we cannot currently predict the occurrence of LVNC and no definitive treatment is yet available, we can provide patients with a better quality of life through aggressive symptomatic and preventive treatment.

Many studies have successfully identified genes associated with cardiomyopathy (118–125), and this information can be employed in genome editing technology (126); while gene therapy will progressively develop in an increasing number of genetic tests, making the future eradication of LVNC promising, researchers are constantly refining genomic and proteomic analyses through animal models. These studies provide ideas for new therapeutic strategies in the future.

However, hopes always coexist with challenges, and the safety and effectiveness of genome editing technology cannot be guaranteed (127–129). Currently, human genome editing therapy technology is still under development. Whether in the experimental design or environment different from the human body, the therapeutic effect of gene therapy on cardiomyopathy is uncertain. Although the experimental data are promising, guaranteeing its effectiveness for practical clinical application is impossible. Moreover, owing to the great unknown of genome therapy, its toxicity and side effects may also be unsustainable. The possibility that gene editing may cause other mutations or symptoms must be considered. Moreover, most current gene therapy strategies are conducted on animal models, and once they are ready to be applied to human bodies, we must ask ourselves about the potential practical and clinical problems; for instance, who is the subject of gene editing, when and under what circumstances, what might be the possible consequences, treatment measures, and expected results. The related human genome editing program must be conducted through constant research and discussion. It is necessary to ensure not only its rationality and effectiveness but also ethical proceeding. Only with the support of a multidisciplinary team of professionals can we advance human genome editing treatment strategies (130, 131).

In conclusion, patients with LVNC of any type should be closely followed up and analyzed because of the conditions’ unknown nature and poor prognosis (132–134). Especially when LVEF function decreases, the prognosis of patients will be worse (28, 70, 135). We also recommend perfect genetic testing for every LVNC patient and their immediate family members, which will be of great importance to advance our research for patients with LVNC.
